# Superior Protective Effects of *in Vitro* Propagated Green Garlic Against Hydrogen Peroxide-induced Cytotoxicity in Human Hepatoma Cells

**DOI:** 10.2478/aiht-2020-71-3399

**Published:** 2020-06-29

**Authors:** Tomislav Vinković, Nada Parađiković, Monika Tkalec Kojić, Gordana Mendaš, Tanja Živković Semren, Valentina Gluščić, Ivana Vinković Vrček, Ivan Pavičić

**Affiliations:** 1 Josip Juraj Strossmayer University of Osijek, Faculty of Agrobiotechnical Sciences Osijek, Osijek, Croatia; 2 Institute for Medical Research and Occupational Health, Zagreb, Croatia

**Keywords:** *Allium sativum* L., alliin, antioxidant activity, glutathione, total phenolic content, total flavonoid content, Allium sativum L., alin, antioksidacijska aktivnost, glutation, ukupni sadržaj fenola, ukupni sadržaj flavonoida

## Abstract

Garlic is a valuable source material for medicines due to its known antitumor, hypolipidaemic, antioxidant, and immunomodulatory effects. This study compares the protective effects of conventionally grown (CG) and *in vitro* propagated garlic (PG) against hydrogen peroxide-induced cytotoxicity in HepG2 cells and their antioxidant activity. Garlic used in this study was obtained by planting garlic cloves or by planting the transplants of PG directly in the field. At the end of the vegetation period, CG and PG were sampled and extracts prepared for the experiment. Compared to conventionally grown garlic bulbs, PG leafy part yielded significantly higher content of polyphenols, flavonoids and alliin, and also showed equal or higher antioxidant activity, measured by the cell viability test, GSH and ROS level. Moreover, PG can be produced in less time (shorter vegetation period) and with significantly less material (cloves). Significantly higher content of alliin, polyphenols, and flavonoids and significantly higher yield of plant biomass in PG has a great potential to become a new production model with improved garlic properties as a medicine material.

## Abstract (hr)

Češnjak je vrijedan izvor ljekovitih sastojaka te je i dalje vrlo raširen, posebice zbog svojih antitumorskih, hipolipidemičkih, antioksidacijskih i imunomodulatornih svojstava. Ovo istraživanje pokazuje protektivni učinak te antioksidacijsko djelovanje konvencionalno uzgojenog i češnjaka uzgojenog iz *in vitro* presadnica. Češnjak korišten u ovom istraživanju dobiven je sadnjom češnjeva (CG) ili *in vitro* presadnica (PG) izravno u tlo. Nakon završetka vegetacije, CG i PG češnjak su uzorkovani te su pripremljeni ekstrakti kako bi se usporedilo njihovo protektivno djelovanje te antioksidacijska aktivnost. Utvrđeno je kako metoda uzgoja koja je rezultirala krajnjim prinosom lukovice kod CG ili nadzemnog dijela kod PG češnjaka značajno utječe na povećanje sadržaja polifenola, flavonoida, antioksidacijski učinak i na sadržaj alina. Također, ispitano je protektivno djelovanje ekstrakata češnjaka na prethodno induciranu citotoksičnost vodikovim peroksidom kod HepG2 stanica. Na temelju testa stanične vijabilnosti, sadržaja GSH i ROS-a utvrđeno je kako PG češnjak može biti jednako ili više učinkovit u svom ljekovitom i antioksidacijskom djelovanju u odnosu na CG češnjak. Nadalje, PG u usporedbi s CG češnjakom može se proizvesti u kraćem vegetacijskom razdoblju, u kojem se ostvaruje veći prinos korištenjem manje originalnog reprodukcijskog materijala (češnjeva). Na kraju, potrebno je istaknuti kako značajno veći sadržaj alina, polifenola i flavonoida, uz istovremeni značajno veći prinos biomase, utvrđen kod PG češnjaka, eventualno donosi novi uzgojni model češnjaka s povećanim sadržajem bioaktivnih komponenti, što poboljšava funkcionalnost češnjaka kao izvora ljekovitih sastojaka.

Garlic (*Allium sativum* L.) has been used as a raw material in traditional medicine of many nations since ancient times and has remained attractive to this day due to its known antitumour, hypolipidaemic, antioxidant, and immunomodulatory effects (1, 2, 3). More recent studies report that these effects mainly originate from bioactive compounds like organosulphuric alliin and allicin, flavonoids, trace minerals, enzymes, and aromatic compounds (1, 4). One of the most important is its potential to treat or prevent cancer due to antioxidant activities of its components.

Consequently, the demand for natural garlic antioxidants continues to rise. One of the ways to meet this demand is *in vitro* propagation of garlic, as it can shorten the vegetation time, increase the reproduction rate, and provide higher yields of biomass and natural antioxidants (5, 6). This study aimed to show the differences in plant biomass yield and biological properties of traditionally grown and *in vitro* propagated garlic and to compare the protective effects of their extracts against oxidative damage in human hepatoma cells. To the best of our knowledge, this is the first study of the kind, and we hoped to find out whether the new propagation model has a potential for production of garlic intended for medicinal use.

## Materials and methods

### Field experiment

Garlic was grown either by planting the cloves of autochthonous garlic cultivar Slavonian winter garlic (cv. Slavonski ozimi; collected in July 2016) directly in the field on 5 November 2016 (conventional garlic – CG) or by propagating garlic (PG) *in vitro* and planting the transplants in the field on 9 March 2017. Plant material was supplied by the Josip Juraj Strossmayer University of Osijek, Faculty of Agrobiotechnical Sciences Osijek, Osijek, Croatia. Field in-row spacing for planting CG was 10 cm and inter-row spacing 35 cm (10x35 cm). PG was planted at 5x20 cm plant spacing. In other words, plant density of CG was 28.5 and of PG 100 plants per square meter. Both CG and PG were grown on 4 m^2^ parcels in quadruplicate following the randomised block design. At the end of the vegetation period on 11 July 2017, which corresponds to the harvest time, when 70 % of the aboveground part of CG is dry. We collected CG bulbs (cloves) and the leafy part of PG, as it did not form true bulbs with cloves and remained green. This was expected due to skipped vernalisation. During the harvest, we recorded the yield (weight of PG leafy part and CG bulbs) as well as the number of leaves per plant (Table 1).

**Table 1:** Differences in yield and overall alliin content between conventional growing and *in vitro* propagation

Garlic PT	Leaves number	Yield per plant (bulbs or leafy part) (g)	Yield per square meter (g)	Alliin yield per square meter (g)
CG	12.57±0.89^a^	38.69±0.73^a^	1102.77±20.89^b^	9.02±0.15^b^

PG	9.89±0.66^b^	30.57±0.42^b^	3056.98±42.14^a^	74.79±0.94^a^

Means ± standard deviation. Different letters ^a,b^ denote significant differences (P<0.05) between CG and PG

### *In vitro* propagation of garlic

*In vitro* propagation of garlic started in November 2016. Healthy and vital garlic cloves collected in July 2016 were peeled and washed under tap water for 15 min. Cleaned cloves were pre-sterilised as described previously (7, 8, 9) with some modifications. Briefly, cloves were immersed in 70 % ethanol for 2 min and then in 2 % sodium hypochlorite with 2 drops of liquid detergent for 20 min for pre-sterilisation. The cloves were then rinsed with sterile distilled water five times and germs explanted from cloves in a microbiological safety cabinet. To minimise potential contamination, germ surface was also sterilised by immersion in 70 % ethanol (Gram-mol, Zagreb, Croatia, CAS 64-17-5) for 1 min and then in a solution of 2 % sodium hypochlorite and two drops of liquid detergent for 10 min. Rinsed with distilled water, the germs were cut vertically in two to three pieces and placed on a Linsmaier & Skoog (LS) medium supplemented with 30 g/L sucrose, 0.1 g/L myo-inositol (HiMedia Laboratories, Mumbai, India), 8 g/L agar (Acros Organics, New Jersey, USA, CAS 9002-18-0), 1 mg/L 6-benzylaminopurine (Acros Organics, CAS 121439-7), and 0.1 mg/L indole butyric acid (Acros Organics, CAS 87-51-4) and cultured in a growth chamber. The explants were sub-cultured several times, each time after three weeks on the same medium, by separating new micro-shoots as new explants. After gaining about 1700 healthy and viable garlic explants, the explants were sub-cultured on rooting LS medium supplemented with 1 mg/L indole butyric acid and 0.1 mg/L 6-benzylaminopurine. Temperature (21 °C±1 °C) and photoperiod (16 hours of light) were the same throughout cultivation. Rooted garlic plantlets were transferred to polystyrene trays with 78 mL cells filled with sterile substrate (Potgrond H, Klasmann-Deilmann GmbH, Geeste, Germany) and placed in a growth chamber for acclimatisation. *Ex vitro* garlic plants were kept under the same light and temperature conditions as those *in vitro*, with relative air humidity maintained at 90 % for the first week and then gradually decreased to constant 60 % and temperature gradually decreased to 15 °C. After one month of acclimatisation, the transplants were put into soil on 9 March 2017 and were about the same size as conventional garlic (CG) planted in November 2016 but with fewer true leaves formed.

### Plant material and sample preparation

CG and PG garlic extracts were prepared after sample collection from the field in July 2017. Garlic powder was prepared as follows: 400 g of fresh and peeled CG garlic cloves and 400 g of fresh PG leafy part were submerged in liquid nitrogen and then lyophilised. The lyophilisate was then ground into fine powder and stored at -30 °C until analysis.

Extraction for the analysis of antioxidant activity, total phenolic content, and total flavonoid content was performed by adding 1 g of freeze-dried garlic powder to 10 mL of 80 % methanol (v/v) and using an ultrasonic bath at 25 °C for 30 min. Then the extracts were filtered through a 0.2 μm pore size nylon membrane filter (Whatman Inc., Piscataway, NJ, USA) and stored at 4 °C in sealed glass test tubes until analysis.

Extraction for high-performance liquid chromatographic (HPLC) determination of alliin was done by adding 1 g of garlic powder to 10 mL of acetonitrile in the Erlenmeyer flask. The flasks were closed, placed in a water bath shaker and shaken at 80 °C for 4 h. The samples were then centrifuged, and their supernatant filtered through a 0.2 μm pore size nylon membrane filter (Whatmann Inc.). Extracts were kept in sealed glass vials at 4 °C until analysis.

### Determination of total phenolic and flavonoid content in garlic plant material

Total phenolic content was determined with the Folin-Ciocalteu method (10) as described by Chen et al. (11). Briefly, CG and PG extracts (100 μL) were diluted with 5.9 mL of distilled water and mixed. Next, 200 μL of Folin-Ciocalteu reagent was added to the mixture and 2 mL of sodium carbonate solution added 1 min later. The mixture was allowed to react at room temperature in the dark for 120 min. The absorbance was measured at 735 nm. Gallic acid was used as a standard, and the results were expressed as mg of gallic acid equivalents (GAE) per gram of garlic powder.

Total flavonoid content was determined as described by Molina-Quijada et al. (12). Briefly, a 0.05 mL of methanolic extract was mixed with 3.65 mL of distilled water and 0.15 mL of a 5 % (w/v) solution of NaNO_2_. After 5 min, 0.15 mL of 10 % (w/v) AlCl_3_ was added to the mixture, kept for another 1 min, and 1 mL of 1 mol/L NaOH was added. Then, the absorbance of samples was measured at 415 nm with a Cecil 9000 UV-visible spectrophotometer (Cecil Instrumentation Services, London UK). Total flavonoid content was expressed as quercetin equivalents, i.e. in milligrams of quercetin per gram of garlic powder. All determinations were carried out in triplicate.

### Determination of garlic plant material antioxidant activity (AOX)

The DPPH free radical (DPPH•) scavenging ability of garlic extracts was determined as described previously (13). Briefly, a 0.3 mmol/L DPPH• stock solution was prepared by dissolving 7.6 mg of DPPH in 100 mL of methanol. Properly diluted CG and PG extracts of alliin (0.1 mL) were mixed with 2 mL of 0.1 mmol/L of DPPH• solution. The mixture was left in the dark for 25 min and the absorbance measured at 517 nm using a Cecil 9000 UV-visible spectrophotometer (Cecil Instrumentation Services). Trolox (6-hydroxy-2,5,7,8-tetramethylchroman-2-carboxylic acid) was used as a standard reference to convert the AOX of each extract solution to the Trolox equivalent antioxidant activity (TEAC). The results were expressed as millimol of TEAC per gram of garlic powder. All determinations were carried out in triplicate.

### HPLC analysis of alliin

Alliin content in CG and PG samples was determined with a HPLC Varian ProStar system equipped with a 230SDM pump, 410 autosampler, and a 330 UV diode-array detector (Varian, Palo Alto, CA, USA). The chromatographic column was a 250x4 mm I.D., 5 μm particle size Gemmini C18 with a 4x3.0 mm I.D. 5 μm particle size guard column (Phenomenex, Torrence, USA). The mobile phase consisted of acetonitrile and water. The isocratic elution was carried out with 10 % acetonitrile and 90 % water in 7 min. The flow rate was 1 mL/min. The amount injected was 100 μL. The UV spectra were recorded from 200 to 400 nm. The working wavelength for quantitative analysis was 210 nm. Compounds in samples were identified by their retention time and UV spectra, which were compared to the known standards. Alliin content was expressed as milligrams of alliin per gram of garlic powder. All determinations were carried out in triplicate.

### Determination of garlic extract protective effects *in vitro*

The human hepatoma cell line HepG2 was purchased from Sigma-Aldrich (St. Louis, MO, USA), cultivated in 1 % glucose Dulbecco’s Modified Eagle’s Medium supplemented with L-glutamine (2 mm), and 10 % foetal bovine serum. Cell cultures were routinely grown in 25 cm^2^ flasks (TPP Techno Plastic Products AG, Trasadingen, Switzerland) in a humidified atmosphere with 5 % CO_2_ at 37 °C until the growth reached the exponential stage. Growth medium was refreshed every three or four days.

To determine the antioxidant activity of garlic extracts, hepatoma cells were exposed to 200 μmol/L H_2_O_2_ (Kemika, Zagreb, Croatia) to induce oxidative stress (14, 15, 16, 17, 18, 19, 20). The protective effect of garlic extracts was evaluated by adding CG and PG extracts to a cell culture medium 20 min after cell exposure to H_2_O_2_. Exposure to garlic extract lasted 24 h at 37 °C. The effects of CG and PG extracts were compared with the antioxidant activity of commercial alliin (S-Allyl-L-cysteine sulphoxide, Sigma-Aldrich) alone. All extracts were dissolved, sterilised by filtering through Whatman 0.2 μm pore size nylon membrane filters, and then added to cells in concentrations below 1000 μg/mL for garlic or 100 μmol/L for commercial alliin, which turned out not be toxic to HepG2 cells in a preliminary 24-hour exposure test, while higher concentrations induced apoptosis (data not shown). These were 10, 100, and 1000 μg/mL for CG and PG extracts and 1, 10, and 100 μmol/L for commercial alliin, prepared by diluting garlic stock solutions with PBS buffer until they corresponded to the concentration of applied alliin alone. Cells exposed to H_2_O_2_ alone (positive control) and non-treated cells (negative control) were included in each experiment.

The metabolic activity and viability of HepG2 cells was assessed using the WST-8 colorimetric assay (CCK-8, Sigma). The cells were seeded in quadruplicate in 96-well tissue culture plates at the concentration of 1x10^5^ cells/mL. After a 48-hour incubation, the cells were treated with 200 μmol/L H_2_O_2_ and after 20 min with different concentrations of CG and PG extracts and alliin for 24 h. At the end of the treatment, the cells were washed with PBS, and 10 μL of WST-8 solution was added to each well (21). The cells were incubated in a CO_2_ incubator at 37 °C for 4 h. Optical density inside the well was determined with a Victor3™ multilabel plate reader (Perkin Elmer, Waltham, MA, USA). Data are expressed as the percentage of absorbance of corresponding positive and negative control.

The level of intracellular reactive oxygen species (ROS) was measured with a Sigma fluorescent 2’,7’-dichlorodihydrofluorescin diacetate (DCFH-DA) probe. All of the measurements were performed in dark sided 96-well microplates. Following the treatment, the cells were washed with PBS and loaded (30 min, 37 °C in the dark) with 50 μmol/L DCFH-DA dye. Fluorescence intensity was detected by Victor3™ multilabel plate reader at an excitation wavelength of 485 nm and an emission wavelength of 535 nm (22). Data are expressed as a percentage of fluorescence of corresponding positive and negative control.

Cell glutathione (GSH) was quantified with a fluorescent monochlorobimane (mBCl) probe. The cells (1x10^5^ cells/mL) were seeded in black 96-well tissue culture plates for 48 h before the treatment. The cells were then exposed to 200 μmol/L H_2_O_2_ for 20 min and then to different concentrations of CG and PG extracts and alliin for 24 h. Followed washing with PBS and incubation with 50 μm mBCl at 37 °C in the dark for 20 min (23). The amount of GSH was analysed using a Victor3™ multilabel plate reader at an excitation wavelength of 355 nm and emission wavelength of 460 nm. Data are expressed as a percentage of fluorescence of the corresponding positive and negative control.

### Statistical analysis

For factorial analysis of variance (ANOVA) and the Tukey HSD test of differences between treatments we used the SAS 9.1 (SAS Institute Inc., Carry, NC, USA) statistical package. The data are reported in figures as means with standard deviations in error bars. Statistical significance was set at P<0.05.

## Results and discussion

The growing techniques (CG and PG) investigated in our study significantly differed in yield, antioxidant activity, and the content of polyphenols, flavonoids, and alliin.

As expected, PG had a significantly higher yield in the leafy part than CG in bulbs. Overall alliin yield per unit area was about eight times higher for PG than CG (Table 1). Except for higher alliin concentration in PG (Figure 2), the main reason for this is significantly higher plant density. Dyduch and Najda (24) reported the same plant density and similar yields of the leafy part of garlic, but they cultivated garlic traditionally by planting cloves or air bulbils to harvest leaves and needed significantly more cloves than we needed for PG in our study.

**Figure 1:**
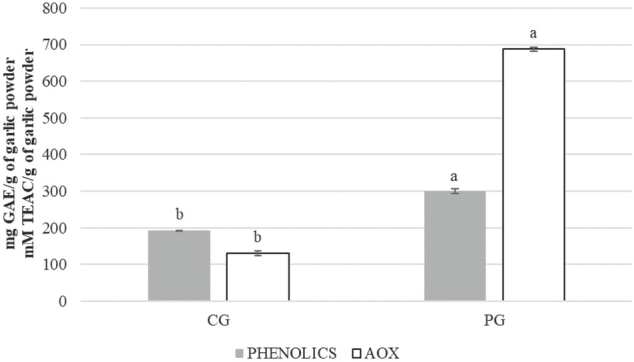
Total garlic extract phenolic content (expressed as mg of GAE per gram of garlic powder) and antioxidant activity (AOX, expressed as mm of TEAC per gram of garlic powder). Values represent means ± standard deviations. Different letters denote significant differences (P<0.05) between CG and PG. GAE – gallic acid equivalent; TEAC – Trolox equivalent antioxidant activity

**Figure 2:**
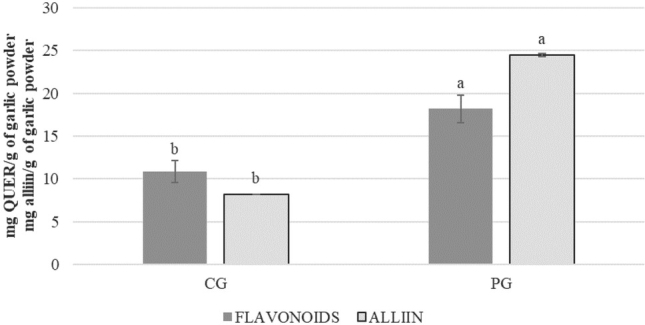
Total flavonoid and alliin content (expressed as milligrams of quercetin and alliin per gram of garlic powder, respectively). Values represent means ± standard deviations. Different letters denote significant differences (P<0.05) between CG and PG

It is well known that antioxidant content (such as polyphenols and flavonoids) in garlic greatly varies with the plant part being analysed as well as genetic, agronomic, and environmental factors (25, 26). In our study, PG leaf extracts had significantly higher levels of polyphenols, flavonoids, and alliin and exerted significantly stronger antioxidant activity (AOX) than CG clove extracts (Figures 1 and 2). Similar was reported by Štajner and Popović (27) for garlic leaves compared to bulbs. They also found significant quantities of carotenoids in garlic leaves and none in the bulbs. In contrast, Chen et al. (11) reported significantly higher polyphenol content in the bulbs of 40 garlic cultivars than the aboveground part, probably due to differences in cultivars and cultivation method than used in our study. However, they also found significantly higher flavonoid content and antioxidant activity in garlic bolts than bulbs of most tested cultivars, which is similar to our findings (Figures 1 and 2).

**Figure 3:**
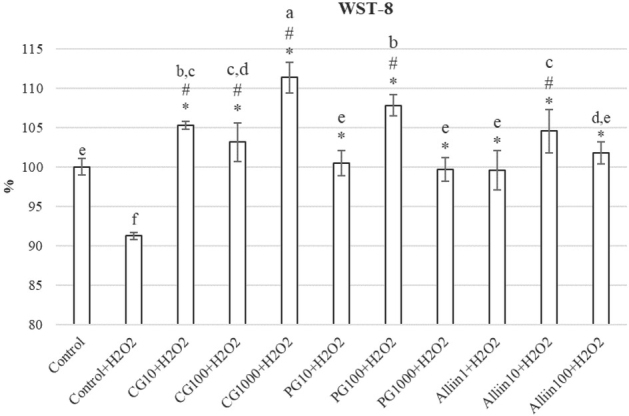
Hepatoma cell viability according to the WST-8 colorimetric assay. Data are expressed as a percentage of absorbance with respect to negative control as a starting point (100 %). Values represent means ± standard deviations. Values marked with the same letters are not significantly different, whereas values marked with different letters are significantly different. # indicates significant difference from negative control and * from positive (H_2_O_2_) control (P<0.05)

Higher polyphenol, flavonoid, and alliin content in our PG extracts suggests that *in vitro* propagation is superior to traditional cultivation as a source of valuable antioxidants, which in turn renders it more effective than CG against oxidative damage, as its components prevent lipid peroxidation, lower ROS generation, and increase the activity of antioxidant enzymes in cells (1, 28). The most beneficial effect for cell viability was achieved with the CG extract at 1000 μg/mL (CG1000), followed by the PG extract at 100 μg/mL (PG100). Overall, the cell viability test (WST-8) showed significant superiority of CG and PG extracts over alliin alone, while all three had significantly higher cell viability than both negative and positive control cells, as expected (Figure 3).

In non-tumour intestinal cells (INT‑407), Mansingh et al. (29) reported that alliin from fresh garlic extract did neither inhibit nor induce cell proliferation. Ghazanfari et al. (30) reported that garlic extract increased cell viability of normal L929 cells after 48 h as well as of HT-29 human colon adenocarcinoma cells. In other words, garlic extracts can have different but mostly positive effects on different cells, which may not be the targeted effect in case of cancer treatment.

**Figure 4:**
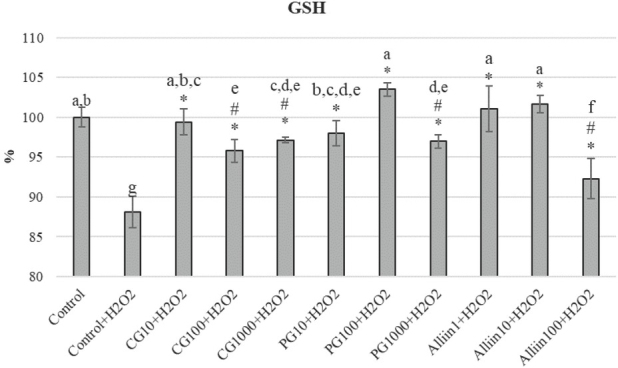
GSH levels determined with the MBCI fluorescent probe. Data are expressed as a percentage of fluorescence of negative control as a starting point (100 %). Values represent means ± standard deviations. Values marked with the same letters are not significantly different, whereas values marked with different letters are significantly different. # indicates significant difference from negative control and * from positive (H_2_O_2_) control (P<0.05)

Figure 4 shows GSH levels in HepG2 cells, which were the highest with PG100 treatment, followed by Alliin1, Alliin10, and CG10 treatments. This indicates that garlic reduces oxidative stress induced by H_2_O_2_ by significantly increasing GSH level.

**Figure 5:**
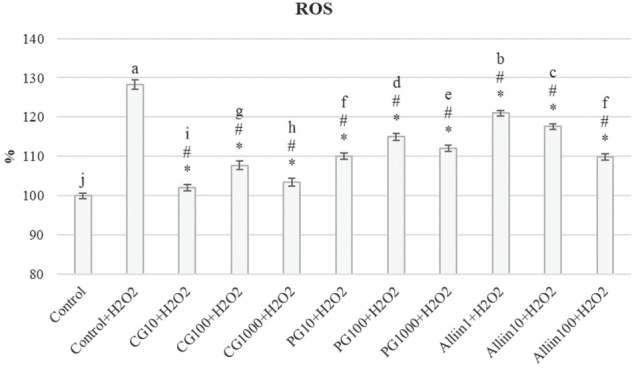
Intracellular ROS levels determined with the DCFH-DA fluorescent probe. Data are expressed as a percentage of fluorescence of negative control as a starting point (100 %). Values represent means ± standard deviations. Values marked with the same letters are not significantly different, whereas values marked with different letters are significantly different. # indicates significant difference from negative control and * from positive (H_2_O_2_) control (P<0.05)

Considering the ROS levels, CG and PG extracts as well as alliin significantly inhibited ROS generation in respect to positive control. The most successful was the CG extract at 10 μg/mL (CG10) (Figure 5).

All of these findings in our study, that is, ROS inhibition and increased cell viability and GSH evidence the protective effect of garlic. Its beneficial stem from a variety of bioactive compounds it contains, organosulphur in particular. According to Yamaguchi and Kumagai (31), the content of the major organosulphur compound alliin in fresh garlic is high and ranges from 3 to 14 mg/g fresh weight and from 20 to 30 mg/g dry weight, which corresponds to alliin content in PG, whereas CG seems to have yielded significantly less alliin (Figure 2).

Chung (32) reports that alliin is a superior superoxide scavenger than other organosulphur compounds and equal to allyl disulphide in hydroxyl scavenging but superior than other sulphur compounds. Namely, there are more than 20 organosulphur compounds in garlic, such as allicin, diallyl trisulphide, and methyl allyl trisulphide, beside the above mentioned, all of which exhibit antioxidant, anticancer, antithrombotic, and antibacterial activity (20, 33, 34). However, in our study lower alliin concentrations (Alliin1, Alliin10) were effective at increasing GSH level but less effective at increasing HepG2 cell viability and lowering ROS compared to CG and PG extracts, which showed a more balanced protective effect. This implies that other bioactive components present in garlic extracts contribute to the protective effect. Anoush et al. (35) reported similar significant superiority of the garlic extract over a single organosulphur compound allicin.

## Conclusion

Our findings suggest that, overall, *in vitro* propagated garlic is superior to the conventionally grown one, as it requires shorter vegetation time, provides higher yields of plant biomass with significantly less input material (cloves), and has a significantly higher content of alliin, polyphenols, and flavonoids. All these benefits highlight the potential of a new production model of garlic for medicinal purposes.
